# Efficacy of PD-1/PD-L1 Inhibitors versus Chemotherapy in Lung Cancer with Brain Metastases: A Systematic Review and Meta-Analysis

**DOI:** 10.1155/2022/4518898

**Published:** 2022-05-20

**Authors:** Xiaojun Yang, Yihong Zeng, Qinquan Tan, Zhihua Huang, Jun Jia, Guanming Jiang

**Affiliations:** ^1^Department of Oncology, Dongguan Institute for Clinical Cancer Research, Affiliated Dongguan People's Hospital, Southern Medical University, 3 Wandao Road South, Dongguan, 523059 Guangdong, China; ^2^Department of Nephrology and Immunology, Dongguan Marina Bay Central Hospital, Dongguan 523900, China

## Abstract

Immune checkpoint inhibitors (ICIs) are widely used to treat local or metastatic lung cancer. However, the efficacy of ICI in patients with brain metastases (BM) from lung cancer is unknown. This study aimed to evaluate the efficacy of PD-1/PD-L1 ICIs compared with chemotherapy for patients with lung cancer with BM. Electronic databases (PubMed, Embase, The Cochrane Library, and Web of Science) were searched. The meta-analysis assessed overall survival (OS) and progression-free survival (PFS) of the PD-1/PD-L1 inhibitors axis and its relationship with pathological type, drug modality, and the treatment line number in patients with BM from lung cancer. We included 694 patients with BM from lung cancer from 11 randomized controlled trials. Statistical analysis showed that compared with chemotherapy, PD-1/PD-L1 inhibitors could significantly prolong OS (hazard ratio (HR) = 0.75, 95%confidence interval (95%CI) = 0.51–0.99) and PFS (HR = 0.65, 95%CI = 0.51–0.80). In the subgroup analysis, ICIs plus chemotherapy improved PFS (HR = 0.60, 95%CI = 0.40–0.80), but not OS (HR = 0.75, 95%CI = 0.30–1.19). The efficacy of ICI monotherapy in patients with BM was significantly different between OS and PFS: OS pooled HR = 0.81 (95%CI = 0.57–1.05) and PFS = 0.78 (95%CI = 0.62–0.94). Among different pathological types, the OS pooled HR was 0.67 (95%CI = 0.39–0.95) for non-small cell lung cancer (NSCLC) and 0.94 (95%CI = 0.56–1.33) for small cell lung cancer (SCLC); the PFS pooled HR was 0.58 (95%CI = 0.39–0.76) for NSCLC and 0.79 (95%CI = 0.65–0.93) for SCLC. Subgroups analysis of treatment line showed that no advantage for OS with ICIs as first-line or subsequent-line therapy, whereas ICIs as first-line (HR = 0.63, 95%CI = 0.53–0.74) and second-line (HR = 0.62, 95%CI = 0.62–0.96) benefitted PFS. This meta-analysis implied that compared with chemotherapy, PD-1/PD-L1 inhibitors significantly improved efficacy treatment of patients with BM from lung cancer. Further studies are needed to confirm the role of ICIs in different pathological types and drug treatment modalities.

## 1. Introduction

Lung cancer is one of the leading causes of human cancer death. According to the latest statistics from the American Cancer Society [1], in the previous ten years (2008–2017), lung cancer death rates have declined quicker than breast, prostate, and colorectal cancers. However, lung cancer still had the highest mortality rate in 2017, surpassing that of breast cancer, prostate cancer, and brain cancer put together. In China, because of the persistent expansion of the number of smokers, around 3,000 individuals are killed by tobacco each day [2]. The central nervous system (CNS) is considered one of the most common sites of lung cancer migration. At diagnosis, nearly 10% of patients with lung cancer have identified brain metastases (BMs), and approximately 40–50% of patients with lung cancer develop new CNS metastases during treatment [3, 4]. Small cell lung cancer (SCLC) has a higher incidence of BMs than non-small cell lung cancer (NSCLC). The prognosis of patients with lung cancer worsens once they develop BMs. Typically, the median overall survival (mOS) time for traditional treatment is 5 months, and the 1-year survival rate is 14% [5]. Additionally, symptoms related to the brain can have an impact on the quality of life of patients.

Currently, the development of lung cancer treatments has led to the survival time of patients with advanced lung cancer being increased to a certain extent. Besides traditional methods like chemotherapy, radiation therapy, surgery, and molecularly targeted therapy that were used in the past, emerging immunotherapeutic agents, such as checkpoint inhibitors, are also showing promising therapeutic results in the treatment of lung cancer brain metastases. The blood-brain barrier prevents the majority of traditional chemotherapy drugs from having the desired therapeutic effect [6, 7], except for temozolomide [8], pemetrexed [9], and topotecan [10]. Historically, radiotherapy and surgical treatment were the benchmarks for the treatment of BMs. Modern molecularly targeted drugs have significantly improved the treatment of mutation-positive NSCLC BMs. According to the FLAURA [11] and AURA3 [12] trials, the molecularly targeted drug osimertinib could delay the progression of BMs, prevented them from occurring and could improve the survival of patients with BMs. In addition to targeted therapies for the epidermal growth factor receptor (EGFR) pathway, small-molecule inhibitors of the ALK receptor tyrosine kinase have achieved spectacular results. The efficacy of alectinib, a tyrosine kinase inhibitor (TKI), was confirmed by the J-ALEX study [13], which found that it could not only control BMs, but also prevented the development of new intracranial lesions. Combining TKIs with other therapies (such as radiotherapy and surgery) has also shown good therapeutic results. On the one hand, surgical intervention could help to obtain pathological tissue to aid diagnosis, and on the other hand, it could rapidly reduce intracranial pressure to relieve symptoms. Radiation therapy is also an effective and widely used treatment for BMs from lung cancer, especially in SCLC. In patients with limited-stage SCLC after a favorable response to systemic therapy, prophylactic cranial irradiation (PCI) decreased the incidence of BMs and improved OS [14, 15]; however, it had a negative impact on cognitive function and quality of life.

Immune checkpoint inhibitors (ICIs) have been approved by the US Federal Drugs Agency (FDA) for numerous indications in lung cancer. Studies have shown that ICIs mainly kill tumor cells by enhancing the peripheral effect of CD8+ T cells in the brain [16, 17]. In the majority of prospective clinical studies of immunotherapy excluded symptomatic BMs and only included patients with asymptomatic or stable BMs after treatment. Numerous clinical trials have demonstrated that checkpoint inhibitors could provide superior efficacy compared with chemotherapy in patients with BM from lung cancer. A phase III clinical trial, the OAK study [18], comparing the efficacy of atezolizumab versus docetaxel has been conducted for NSCLC. A total of 850 patients with advanced NSCLC were randomly assigned to the atezolizumab and docetaxel groups, which included 61 and 62 patients with BMs, respectively. In the exploratory analysis of BMs, the median OS of atezolizumab *vs*. docetaxel was 16 months and 11.9 months (the difference was not statistically significant); the median time to new cranial lesions was not reached by atezolizumab, and the median time for docetaxel was 9.5 months. Nevertheless, the efficacy of ICIs for BMs from lung cancer remains controversial. Results from the KEYNOTE-024 study [19] demonstrated that a longer median progression free survival (PFS) was observed in the pembrolizumab group (*n* = 18) than in the chemotherapy group (*n* = 10) (*hazard* *ratio* (*HR*) = 0.55; 95% confidence interval (CI) 0.2–1.56); however, the differences between the groups were not statistically significant. Pooling data from the CheckMate017, CheckMate057, and CheckMate063 studies (Nivolumab phase 2 trial in NSCLC), patients with pretreated BMs survived longer with nivolumab (8.1 months; 95%*CI* = 5.0–11.6) than with docetaxel (6.2 months; 95%*CI* = 4.4–9.2) [20] .

Conventional chemotherapy has reached a bottleneck in BMs derived from driver-negative lung cancer, especially in those who are unable to undergo surgery or radiotherapy; therefore, new treatments are urgently needed to tackle this challenge. ICIs are one of the most promising therapeutic approaches, and various studies have demonstrated the initial efficacy of anti-programmed cell death 1 (PD-1) or anti-programmed cell death 1 ligand 1 (PD-L1) monoclonal antibodies in BMs from lung cancer; however, there have also been contrasting results. Whether PD-1/PD-L1 ICIs are better than chemotherapy in patients with BMs from lung cancer remains uncertain. Therefore, we conducted this meta-analysis to explore whether PD-1/PD-L1 ICIs affect the survival of patients with BMs from lung cancer.

## 2. Materials and Methods

### 2.1. Literature Search Strategies

This study was registered with the International Prospective Register of Systematic Reviews (PROSPERO) before it was conducted (number CRD42021228095). Two independent researchers selected relevant studies published between January 1, 2000, and July 1, 2021, by searching PubMed, Embase, The Cochrane Library, and Web of Science. The search was performed using the following keywords: “Lung Neoplasms,” “Lung Carcinoma,” “Lung Cancer,” “Non-Small-Cell Lung,” “Small Cell Lung Cancer,” “NSCLC,” “SCLC,” “Brain metastases,” “Immunotherapy,” “Checkpoint Inhibitors,” “Programmed Cell Death 1 Receptor,” “Programmed Cell Death 1 Ligand 1”, PD-1,” “PD-L1,” Randomized Controlled Trial,” “Atezolizumab,” “Durvalumab,” “Pembrolizumab,” “Nivolumab,” “Camrelizumab,” “Sintilimab,” “Tislelizumab,” and “Toripalimab.”

### 2.2. Study Selection and Data Extraction

The inclusion and exclusion criteria were as follows: In the inclusion criteria, (1) NSCLC or SCLC confirmed by cytological or pathological examination and confirmed as stage IV lung cancer by imaging or other clinical examination; (2) phase II or III clinical randomized controlled trial (RCT); (3) the study protocol was PD-1/PD-L1 monotherapy or combination therapy versus chemotherapy; (4) and there were PFS or OS outcome data with baseline BM in the study. The exclusion criteria are as follows: (1) Repeated research and (2) retrospective research, case report, meta-analysis, and review. A study with multiple publications was analyzed based on its most recent publication. The studies were screened by two authors, and when differences arose, we resolved them through negotiation or according to the decision of a third author. The primary endpoints of the study were PFS and OS for the population with BM in lung cancer. The extracted data include trial name, author, publication year, stage of trial, tumor histology, treatment line, trial drugs (intervention/comparison), number of patients with BM, criteria for allowing inclusion of patients with BM, primary outcome, and 95% CI.

### 2.3. Quality Assessment

Researchers independently assessed the risk of bias in each study, according to the Cochrane Risk of BiasTool [21], which accounts for sequence generation, allocation concealment, blinding, incomplete data, and selective reporting. We classified the studies as low risk, high risk, and unclear risk.

### 2.4. Statistical Analysis

The statistical analysis used STATA 14.0 (StataCorp LLC, College Station, TX, USA) and RevMan 5.4 software to perform statistical analysis on the data. The comparison between the PD-1/PD-L1 immunotherapy group and the chemotherapy group used the HR as the effective index, and each effect size was given its point estimate and 95% CI. The I^2^ statistic in the heterogeneity test was used to detect the statistical heterogeneity of the included studies. *P* ≥ 0.10 and I^2^ ≤ 50% indicated that there was no statistical heterogeneity among the studies, and the fixed effects model was used for analysis. *P* < 0.10 or I^2^ > 50% indicated that there was statistical heterogeneity among the studies; thus, the random-effects model was used for analysis. If there was statistical heterogeneity between the two groups, the source of the heterogeneity was analyzed, and subgroup analysis was conducted for factors that might cause heterogeneity. If the heterogeneity was too great to analyze the source of the heterogeneity, a descriptive analysis was performed. *P* ≤ 0.05 indicated that the difference was statistically significant.

### 2.5. Evaluation of the Risk of Bias

The risk of bias of the RCTs evaluated the quality and bias of the included literature according to the standards recommended by the Cochrane Handbook version 5.2, including the correct randomization method, whether to use blinding, patient selection, concealment of random allocation plans, missing data reporting, and selectivity We reported the research results and other sources of bias in seven areas, and Begger's test was used to assess publication bias.

## 3. Results

### 3.1. Characteristics of the Eligible Studies

**Figure 1:**
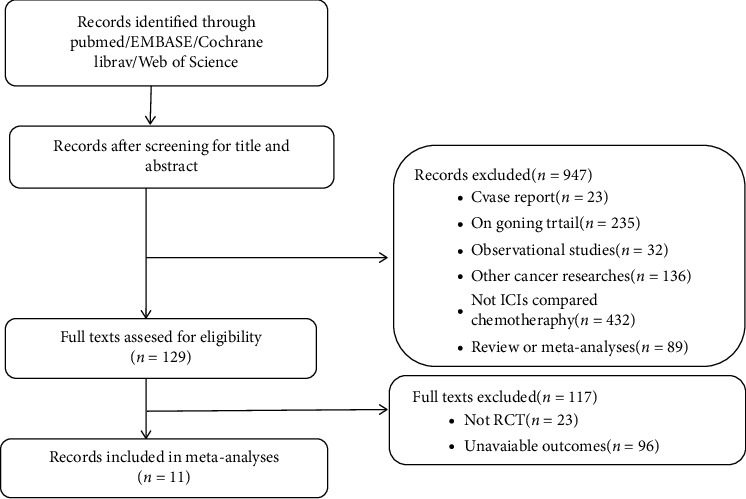
Flow chart of the literature search.

**Figure 2:**
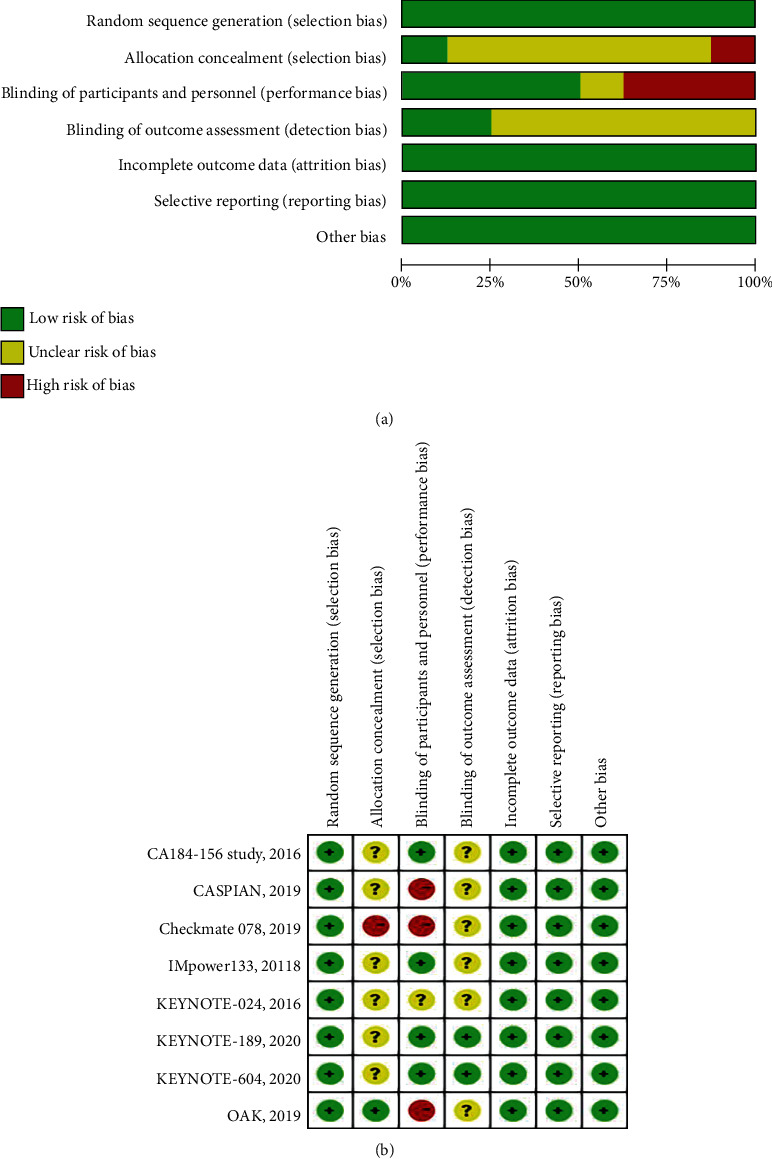
Assessment of risk of bias. (a) Methodological quality graph: authors' judgment about each methodological quality item presented as percentages across all included studies. (b) Methodological quality summary: authors' judgment about each methodological quality item for each included study, “+” low risk of bias; “?” unclear risk of bias; “-” high risk of bias.

In total, the search strategy identified 11 RCTs in the meta-analysis (Figure 1). Of the 694 patients enrolled, 321 were randomly assigned to receive ICIs (monotherapy or combination therapy), and 373 were randomly assigned to receive chemotherapy. The baseline clinical characteristics of the eleven studies [18, 19, 22–30] are summarized in Table 1. Three of studies (CheckMate057, CheckMate078, and OAK) included patients who had previously received one to two cytotoxic chemotherapy regimens, while eligible patients were chemotherapy-naïve in the other eight studies. PD-1 inhibitors were applied in eight studies, while PD-L1 inhibitors were used in three studies (IMpower133, CASPAIN, OAK). Regarding pathological types, three studies (IMpower133, CASPAIN, KEYNOTE-604) included histology of SCLC, while the other eight studies were NSCLC.5 studies (IMpower133, CASPAIN, KEYNOTE-189, OAK, and ORIENT-11) that recruited only patients with asymptomatic NSCLC BMs. Seven studies applied PD-1/PD-L1 inhibitors combined with chemotherapy *vs*. chemotherapy alone, while ICI monotherapy was compared with chemotherapy alone in four studies (KEYNOTE-024, CheckMate057, CheckMate078, and OAK). In one study (ONO-4538-52/TASUKI-52), a PD-L1 inhibitor was combined with Bevacizumab plus chemotherapy, compared with chemotherapy alone. We utilized the Cochrane Collaboration's tool to evaluating the risk bias to determine the quality and potential biased nature of studies (Figure 2).

### 3.2. Benefits of OS and PFS for the Regime of PD-1/PD-L1 Inhibitors vs. Chemotherapy in Patients with BM

**Figure 3:**
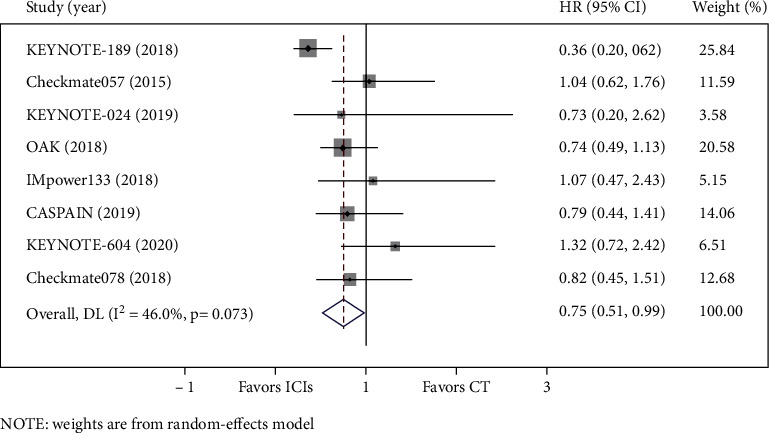
Forest plot of OS between the immune checkpoint inhibitors (ICIs) group and the chemotherapy (CT) group for lung cancer with BM.

**Figure 4:**
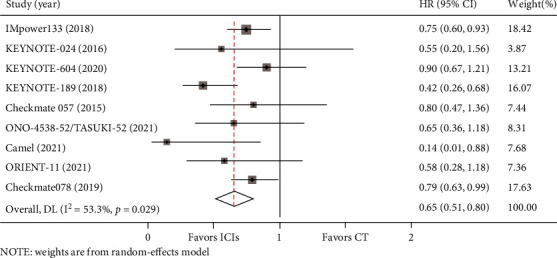
Forest plot of PFS between immune checkpoint inhibitors (ICIs) group and chemotherapy (CT) group for lung cancer with BM.

In the included studies, OS data for BM from lung cancer was reported in eight studies, and PFS data was available in nine studies. Compared with chemotherapy, there was a trend for the PD-1/PD-L1 axis to improve OS for BM from lung cancer (HR = 0.75, 95%CI = 0.51–0.99) (Figure 3). Meanwhile, PD-1/PD-L1 inhibitors contributed to significantly longer PFS than chemotherapy for patients with BM (HR = 0.58, 95%CI = 0.38–0.79) (Figure 4). Heterogeneity was observed for OS (I^2^ = 46.0%, *P* = 0.073) and PFS (I^2^ = 53.3%, *P* = 0.029) in such patients.

### 3.3. Subgroup Analyses by Histological Subtypes

**Figure 5:**
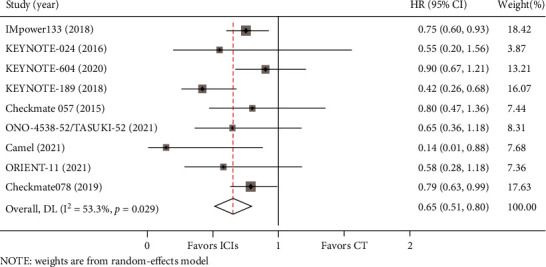
Subgroup analyses of OS by histology between immune checkpoint inhibitors (ICIs) group and chemotherapy (CT) group for lung cancer with BM.

**Figure 6:**
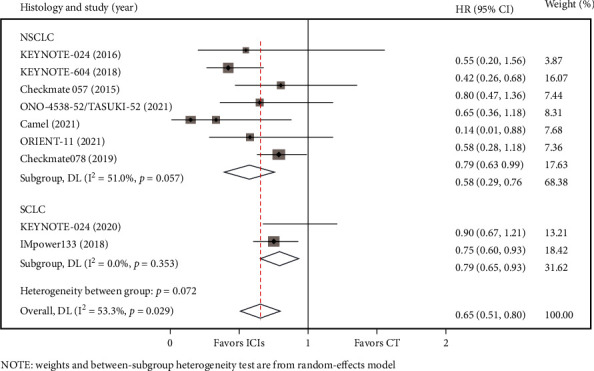
Subgroup analyses of PFS by histology between the immune checkpoint inhibitors (ICIs) group and the chemotherapy (CT) group for lung cancer with BM.

Based on the pathological types of cancers in the included studies, NSCLC and SCLC could be distinguished. According to the results of five studies, heterogeneity in the NSCLC group (*I*2 = 46.0%, *P* = 0.073) was moderate. Based on the above heterogeneity test results, the random-effects model was applied. We observed an OS advantage for patient with NSCLC with BMs (*HR* = 0.67, 95%*CI* = 0.39–0.95) (Figure 5). Meanwhile, in the SCLC subgroup, three RCTs were involved, and the heterogeneity test was I^2^ = 0.0% (*P* = 0.548), and the pooled HR for OS was 0.94 (95%CI = 0.56–1.33) (Figure 5). As shown in Figure 6, seven studies reported PFS data for NSCLC, and for SCLC, only two RCTs reported available PFS data. These subgroup meta-analysis results indicated that PD-1/PD-L1 inhibitors could reduce the risk of disease progression for NSCLC (HR = 0.58, 95%*CI* = 0.39–0.76) with moderate heterogeneity (I^2^ = 51.0%, *P* = 0.057) using a random effects model, and the pooled HR for PFS in SCLC was 0.79 (95%CI = 0.65–0.93) with low heterogeneity (I^2^ = 0.0%, *P* = 0.353).

### 3.4. Subgroup Analyses by Drug Modality

**Figure 7:**
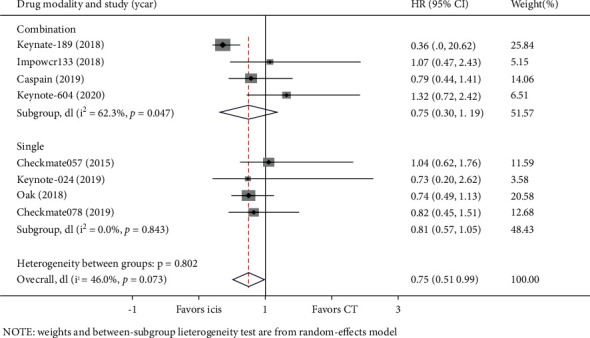
Subgroup analyses of OS by drug modality between the immune checkpoint inhibitors (ICIs) group and the chemotherapy (CT) group for lung cancer with BM.

**Figure 8:**
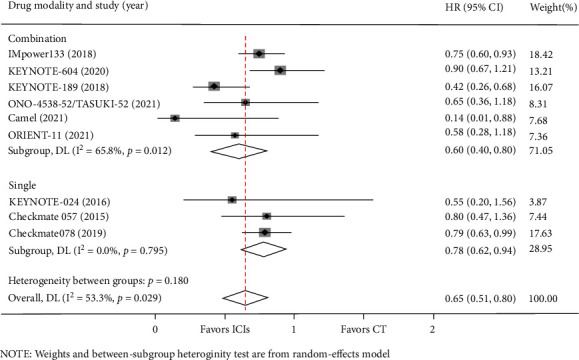
Subgroup analyses of PFS by drug modality between the immune checkpoint inhibitors (ICIs) group and the chemotherapy (CT) group for lung cancer with BM.

In the subgroup analysis of the PD-1/PD-L1 drug modality (combination therapy or monotherapy), we found that four studies used immune monotherapy, with low heterogeneity (I^2^ = 0.00%, *P* = 0.843). Compared with chemotherapy, there was no OS advantage with PD-1/PD-L1 immune checkpoint inhibitors alone in lung cancer BMs (HR = 0.81, 95%CI = 0.57–1.05) (Figure 7). Four studies used PD-1/PD-L1 inhibitors combined with chemotherapy, and the heterogeneity between the groups was moderate (I^2^ = 65.8%, *P* = 0.0012) (Figure 7). The results of this meta-analysis using a random-effects model revealed that the ICI combination therapy versus chemotherapy was not significantly associated with superior OS for patients with BM from lung cancer (HR = 0.75, 95%CI = 0.30–1.19) (Figure 7). Furthermore, six studies reported PFS data on ICI combination therapy, while three studies reported PFS data on ICI monotherapy. In the ICI monotherapy group, the results indicated that compared with chemotherapy, the pooled HR for PFS was 0.78 (95%CI = 0.62–0.94) with low heterogeneity (I^2^ = 0.00%, *P* = 0.795) (Figure 8). Interestingly, when combined with chemotherapy, ICIs could extend PFS (HR = 0.60, 95%CI = 0.40–0.80), and the heterogeneity between the groups was moderate (I^2^ = 65.8%, *P* = 0.012) (Figure 8).

### 3.5. Subgroup Analyses by Treatment Line

**Figure 9:**
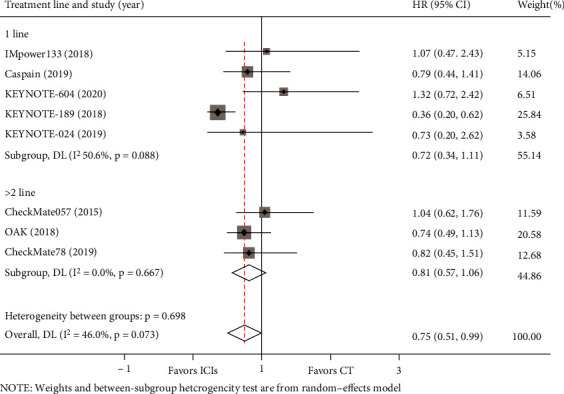
Subgroup analyses of OS by treatment line between the immune checkpoint inhibitors (ICIs) group and the chemotherapy (CT) group for lung cancer with BM.

**Figure 10:**
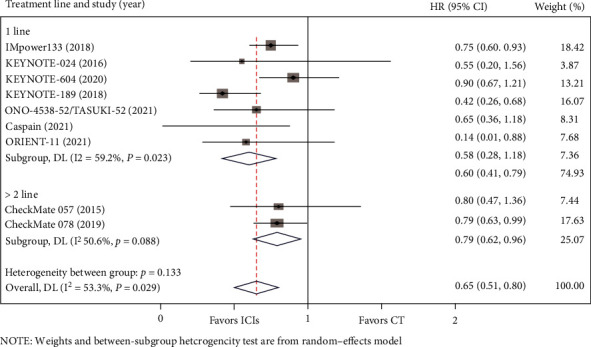
Subgroup analyses of PFS by treatment line between the immune checkpoint inhibitors (ICIs) group and the chemotherapy (CT) group for lung cancer with BM.

We conducted a subgroup analysis of treatment lines in the included studies. Of the eight studies for which OS data was available, five studies included populations with advanced lung cancer that had not received any previous systemic therapies, and the remaining three studies included populations that had undergone at least first-line treatment. According to the results shown in Figure 9, first-line (HR = 0.72, 95% = 0.34–1.11) or subsequent-line (HR = 0.81, 95%CI = 0.57–1.06) treatment with ICIs for lung cancer with BM showed no OS advantage. Of the 8 included studies, 3 were second-line treatments, and the others were first-line treatments. As shown in Figure 10, a superior PFS advantage was achieved in both first-line (HR = 0.63, 95%CI = 0.53–0.74) and second-line (HR = 0.62, 95%CI = 0.62–0.96) treatment with ICIs.

### 3.6. Publication Bias Assessment

**Figure 11:**
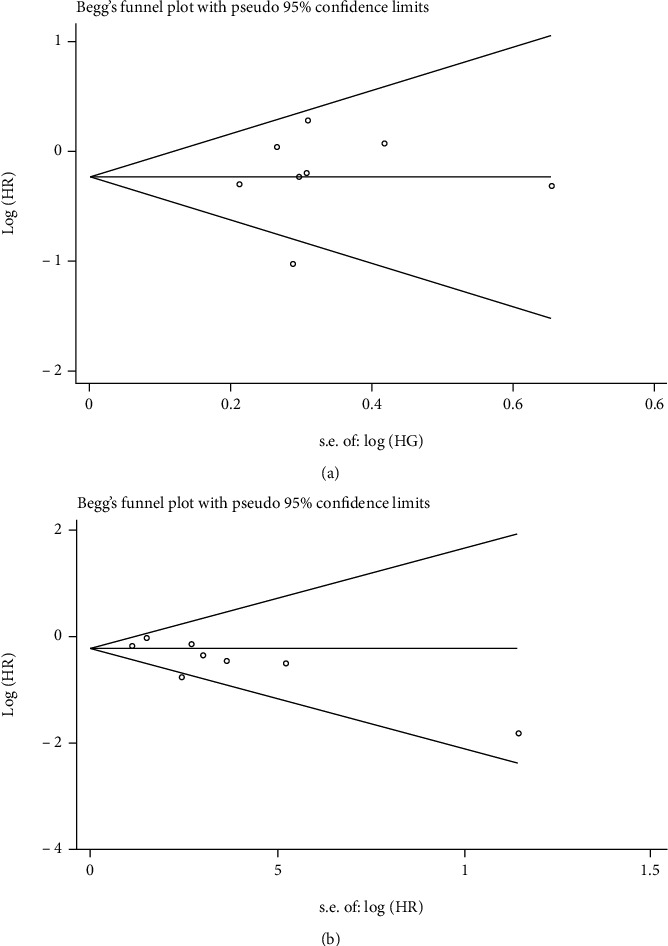
Funnel plots assessing the publication bias using Begg's rank correlation test. (a) OS and (b)PFS.

Publication bias analysis of the included literature using the Begger's test showed no significant publication bias for either OS (*P* = 0.54) or PFS (*P* = 0.11) (Figure 11).

## 4. Discussion

According to our findings, this meta-analysis was a comprehensive evaluation of PD-1/PD-L1 ICI versus chemotherapy for lung cancer with BMs based on the latest research results. To the best of our knowledge, that was the first meta-analysis to explore the efficacy of PD-1/PD-L1 inhibitors in BMs from small cell lung cancer. This meta-analysis demonstrated that PD-1/PD-L1 inhibitors were significantly associated with a 35% reduction in the risk of progression or death compared with chemotherapy for advanced lung cancer with BMs. Meanwhile, there was a significant trend toward increased OS compared with chemotherapy. In the subgroup analysis, there was a greater benefit in NSCLC, with a 33% reduction in deaths. Regrettably, the OS benefit was not better than chemotherapy in SCLC. However, PD-1/PD-L1 inhibitors enhanced PFS in both SCLC and NSCLC. In the subgroup analysis of the medication regimen, PD-1/PD-L1 inhibitor combination therapy and monotherapy was not superior to chemotherapy in OS. As an alternative, PD-1/PD-L1 axis monotherapy and combination therapy offered better PFS compared with chemotherapy in patients with BM. A subgroup analysis based on the treatment line revealed that the OS benefit was independent of the number of prior systemic therapies, yet ICIs improved PFS benefit compared with chemotherapy, both in first-line and second-line treatment.

The CNS has long been considered an immune suppressive environment because of the blood-brain barrier and its minimal lymphocytic infiltration [31, 32]. Furthermore, parenchymal cells in the brain also secrete immunosuppressive factors like indoleamine 2,3-dioxygenase (IDO) [33] and transforming growth factor beta (TGF-*β*) [34]. In the presence of brain metastases, the intracranial immune microenvironment is altered, most notably by a widespread infiltration of activated lymphocytes, including both cytotoxic T cells and immunosuppressive or depleted T cells [35, 36], in addition to a large infiltration of monocyte s [37]. This change in the immune microenvironment provides the basis for PD-1 or PD-L1 therapy. Studies revealed that the increase in tumor response was not caused by an expansion in the number of intracranially infiltrating lymphocytes, but rather to the transport of extracranial T cells (mainly CD8+ T cells) into the CNS to complete the killing effect [38]. This review focuses on the efficacy of immune combination chemotherapy in BMs, and we briefly identify the mechanisms involved. The hypothesis that immunotherapy combined with chemotherapy is synergistic is widely accepted, mainly because cytotoxic drugs can release immunogenic tumor antigens by killing tumor cells and induce upregulation of PD-L1 expression [39], which provides a rationale for PD-1 or PD-L1 therapy. In particular, ICIs lead to an increase in the permeability of the blood-brain barrier (BBB), which helps chemotherapeutic agents penetrate into the brain. According to the above mechanisms, it is theoretically possible that immunotherapy combined with chemotherapy could show improved efficacy against BMs from lung cancer.

There is increasing evidence that ICIs are effective in treating lung cancer BMs. An Italian retrospective study (an expanded access program, EPA) evaluated Nivolumab in the second-line treatment of the BMs, and the intracranial control rate reached 39% [40]. Goldberg reported that the effective rate of Pembrolizumab in the treatment of BMs was 33% (6/18) [41]. There is a limited amount of real-world data on ICI treatment of SCLC with BMs; however, the CASPIAN study [23] showed that immune checkpoint therapy was very promising. A previous meta-analysis [42] reported data on the efficacy of NSCLC with BM, which included three RCTs (Keynote024, OAK, and Keynote189). The results showed that NSCLC with BM could benefit from PD-1 inhibitors (OS HR = 0.43, 95%CI = 0.27–0.69). In the subgroup analysis, compared with ICIs combined with chemotherapy, ICI monotherapy did not bring benefits to patients (HR = 0.71, 95%CI = 0.48–1.04, P = 0.082). Results from the pooled analysis of the lung cancer KEYNOTE series also suggest that pembrolizumab plus chemotherapy (KEYNOTE-021, 189, and 407) showed an improvement in survival, regardless of whether the BM was present at baseline [43]. According to our meta-analysis, PD-1/PD-L1 inhibitors offered OS benefits over chemotherapy for patients with BMs at baseline, which is consistent with previous studies. In contrast to previous findings, the combination of PD-1/PD-L1 checkpoint inhibitors with conventional therapy did not provide an OS benefit. The difference in the results might because of the inclusion of more studies and varying sample sizes. In a retrospective study [44], 77 patients with NSCLC with BM who received either immune combination chemotherapy or monotherapy were included. The results of subgroup analysis revealed that chemotherapy combined with ICIs led to a significantly superior OS rate compared with ICIs alone. The sample sizes in these prior studies were small, and therefore their results need to be interpreted with caution. With regard to the type of pathology, in particular, SCLC with BMs, a total of three RCTs were included in our analysis. The results from the subgroup analysis indicated that ICIs reduced the risk of progression or death by 21% compared with chemotherapy, with no significant difference between the two treatment regimens for OS. Similarly, a lack of benefit of ICIs plus chemotherapy versus chemotherapy alone in patients for SCLC with BM at baseline (*HR* = 1.14, 95%CI = 0.87–1.50) has been reported [45]. Based on the current results, ICIs were not as effective in BMs from SCLC compared with those from NSCLC. One possible reason for this is that SCLC does not attract as many activated T lymphocytes into the brain as NSCLC. Also, there are significant differences in the composition of the tumor microenvironment in NSCLC and SCLC BMs. Additionally, we PCI for patients with SCLC who have achieved a complete or partial response. Not all of the included three clinical studies of SCLC allowed PCI in patients with SCLC who achieved remission at the end of induction chemotherapy, which might have led to intracranial progression in a proportion of patients during ICI maintenance therapy. More prospective studies are needed to explore whether PCI can enhance the efficacy of immunotherapy in SCLC BMs. Most of the current clinical studies on immunotherapy include patients with asymptomatic or treated stable BMs, leaving a proportion of patients outside the scope of immunotherapy. In a retrospective study, the clinical efficacy of immunotherapy for patients with active BMs was explored. Researchers found that PD-1/PD-L1 antibody monotherapy did not reduce the risk of BMs in patients with NSCLC (*HR* = 1.87; 95%CI = 1.13–3.11) [45]. 255 patients with BMs were included in a multicenter retrospective study of advanced NSCLC (39.2% of the patients had active BMs, 14.3% had symptomatic BMs, and 29.4% were prescribed corticosteroids). The intracranial response rate (iCRR) for patients with active BMs (*n* = 73) was 27.3%, and there was a higher incidence of intracranial progression of active BM compared with that of stable BM (54.2% versus 30%, *P* < 0.001) [46]. There are no uniform criteria for the selection of patients with BMs for enrolment; therefore, we did not further explore the difference in efficacy between immunotherapy for active and stable BMs.

Our study had the following limitations: First, the study only analyzed the OS and PFS results of lung cancer BMs, and did not further analyze intracranial efficacy, which can more accurately compare the therapeutic advantages ICIs with those of chemotherapy in lung cancer BMs. Second, we only assessed the efficacy of PD-1/PD-L1 monoclonal antibodies in combination with chemotherapy or single-agent compared with conventional chemotherapy in BMs. Studies have shown a good response rate when ICIs are used in combination with radiotherapy [47, 48] and double immunotherapy [49] for BM. Third, the included studies were mostly asymptomatic or stable lung cancer BMs after treatment. In the real world, more patients have symptomatic BMs; therefore, in clinical practice, we need to be more cautious in interpreting the research results. Besides, the number of included studies was small, and the data sources of the included studies were taken mostly from the subgroup analysis. Therefore, more clinical studies are needed to further confirm the accuracy of the results. Lastly, there was a certain risk of bias because the treatment group and the control group were directly and inevitably imbalanced in terms of confounding factors. Future clinical studies should include patients with active BMs, explore the efficacy of immunotherapy for lung cancer BMs, and identify predictive biomarkers.

## 5. Conclusion

In conclusion, PD-1/PD-L1 checkpoint inhibitors, compared with chemotherapy, significantly prolonged OS and PFS in patients with BM from advanced lung cancer. Our study implied that PD-1/PD-L1 inhibitors could be a therapeutic option for BM, which is related to poor prognosis of lung cancer.

## Figures and Tables

**Table 1:** Characteristics of included studies.

Author	Trial name	Year	Treatment line	Histology	Eligible criteria for BMs	Intervention	Comparison	No. of brain metastases	Media PFS, (months)	Media OS, (months)
Horn, L.	IMpower133	2018	1	SCLC	Asymptomatic, treated	Atezolizumab + EC	Placebo + EC	17/18	5.2 VS 4.3	12.3 VS 10.3
Paz-Ares, L.	CASPAIN	2019	1	SCLC	Asymptomatic, treated	Durvalumab + EC/EP	Etoposide and EC/EP	28/27	5.1 VS 5.4	13.0 VS 10.3
Rudin, Charles M.	KEYNOTE-604	2020	1	SCLC	Untreated	Pembrolizumab + EC/EP	Placebo + EC/EP	33/22	4.5 VS 4.3	10.8 VS 9.7
Reck, M.	KEYNOTE-024	2016	1	NSCLC (NSQ/SQ)	Treated	Pembrolizumab	Platinum-based chemotherapy	18/10	10.3VS6.7	30.2 VS 14.2
Gadiel	KEYNOTE-189	2018	1	NSCLC (NSQ)	Asymptomatic	Pembrolizumab/AP	Placebo + PC/AP	73/35	8.8 VS 4.9	22.0 VS 10.7
Gadiel	OAK	2018	2	NSCLC (NSQ/SQ)	Asymptomatic, treated	Atezolizumab	Docetaxel	61/62	2.8 VS4.0	16.0 VS 11.9
H. Borghaei	CheckMate 057	2015	2	NSCLC (NSQ/SQ)	Treated, stable	Nivolumab	Docetaxel	34/34	2.3 vs 4.2	12.2 VS 9.4
Yi-Long Wu	CheckMate 078	2018	2	NSCLC (NSQ/SQ)	Treated, stable	Nivolumab	Docetaxel	45/27	2.8 vs 2.8	12.0 VS 9.6
S. Sugawara	ONO-4538-52/TASUKI-52	2021	1	NSCLC (NSQ/SQ)	Treated, stable	Nivolumab bevacizumab + TP	Bevacizumab + TP	36/41	12.1VS 8.1	25.4VS24.7
Caicun Zhou,	CameL	2021	1	NSCLC (NSQ/SQ)	Untreated	Camrelizumab + TP	Paclitaxel + TP	10/5	11.3 VS 8.3)	NR VS 20.9
Yunpeng Yang	ORIENT-11	2020	1	NSCLC (NSQ/SQ)	Asymptomatic	Sintilimab + PP	Placebo	36/22	8.9 VS 5.0	NR VS NR

NSCLC, non-small cell lung cancer; SCLC, small cell lung cancer; NR, not reported; NSQ, nonsquamous; SQ, squamous; EC, etoposide and carboplatin; EP, etoposide + cisplatin; PC, pemetrexed + carboplatin; AP, pemetrexed + cisplatin; PP, pemetrexed + platinum; TP, paclitaxel + carboplatin.

## Data Availability

The data used to support the findings of this study are included in the article. Data and materials from this study are available on request from the corresponding author.
